# MEK1/2 inhibition prevents DENV and ZIKV infection via disrupting the cytoskeletal vimentin cage required for viral replication

**DOI:** 10.1093/jmcb/mjaf037

**Published:** 2025-10-25

**Authors:** Yuhan Huang, Jiageng Lu, Shuzhi Cui, Shuangshuang Zhao, Shengming Sun, Yaming Jiu

**Affiliations:** Unit of Cell Biology and Imaging Study of Pathogen Host Interaction, Key Laboratory of Molecular Virology and Immunology, Shanghai Institute of Immunity and Infection, Chinese Academy of Sciences, Shanghai 200031, China; University of Chinese Academy of Sciences, Beijing 100049, China; Unit of Cell Biology and Imaging Study of Pathogen Host Interaction, Key Laboratory of Molecular Virology and Immunology, Shanghai Institute of Immunity and Infection, Chinese Academy of Sciences, Shanghai 200031, China; School of Life Science and Technology, ShanghaiTech University, Shanghai 201210, China; Unit of Cell Biology and Imaging Study of Pathogen Host Interaction, Key Laboratory of Molecular Virology and Immunology, Shanghai Institute of Immunity and Infection, Chinese Academy of Sciences, Shanghai 200031, China; Unit of Cell Biology and Imaging Study of Pathogen Host Interaction, Key Laboratory of Molecular Virology and Immunology, Shanghai Institute of Immunity and Infection, Chinese Academy of Sciences, Shanghai 200031, China; University of Chinese Academy of Sciences, Beijing 100049, China; Shanghai Institute of Materia Medica, Chinese Academy of Sciences, Shanghai 201203, China; International Research Center for Marine Biosciences, Shanghai Ocean University, Shanghai 201306, China; Unit of Cell Biology and Imaging Study of Pathogen Host Interaction, Key Laboratory of Molecular Virology and Immunology, Shanghai Institute of Immunity and Infection, Chinese Academy of Sciences, Shanghai 200031, China; University of Chinese Academy of Sciences, Beijing 100049, China; School of Life Science and Technology, ShanghaiTech University, Shanghai 201210, China

**Keywords:** MEK1/2, vimentin, viral infection, anti-viral effect

## Abstract

*Flaviviridae* Dengue virus (DENV) and Zika virus (ZIKV) have posed significant threats to global public health in the past decades. Despite extensive study on therapeutic strategies against these viruses, effective treatment options are still lacking. Within host cells, the cytoskeletal vimentin intermediate filament network facilitates viral replication during DENV and ZIKV infection by shrinking and forming a cage-like structure. Our previous work indicated that MEK1/2 inhibitors can induce the dispersion of vimentin, but their potential impact on flavivirus infection remains unclear. Here, we observed that the MEK1/2 signaling pathway is activated in host cells infected with DENV and ZIKV. Treatment with MEK1/2 inhibitors significantly impaired the replication of both viruses. Further mechanistic studies revealed that MEK1/2 inhibitors prevent viral infection by promoting the dispersion of intracellular vimentin network, thereby disrupting the cytoskeletal structure required for viral replication. Our findings not only expand the understanding of vimentin regulatory mechanisms from a cellular biology perspective but also provide a new perspective on MEK1/2 inhibition as a potential anti-DENV and anti-ZIKV strategy.

## Introduction

Dengue virus (DENV) and Zika virus (ZIKV), both belonging to the *Flaviviridae* family of single-stranded positive-sense RNA enveloped viruses, are transmitted by the Aedes aegypti mosquito and have garnered global public health attention due to their potential for vertical transmission (Roy and Bhattacharjee, 2021). The structural similarities between DENV and ZIKV, along with their frequent co-circulation in endemic regions, lead to cross-reactive antibodies and co-infections (Avilés-Vergara et al., 2020). As members of the *Flaviviridae* family, DENV and ZIKV share remarkable similarities in their life cycles within host cells. The replication phase of DENV and ZIKV is particularly critical, involving interactions between multiple non-structural proteins (NS proteins) and host factors. These interactions lead to alterations in the endoplasmic reticulum membrane structure, forming a replication complex that facilitates the replication of the viral genome (Coldbeck-Shackley et al., 2020). Currently, there are no specific therapeutic measures for DENV and ZIKV, which underscores the importance of identifying and inhibiting the common signaling pathways triggered during host infection by both.

Vimentin is a type III intermediate filament protein essential for maintaining cellular integrity, with its dynamic rearrangement playing a crucial role in cell migration, signaling transduction, and stress response (Danielsson et al., 2018). Recent studies have increasingly recognized vimentin’s role in pro-viral pathogenesis. During infections with viruses such as severe acute respiratory syndrome coronavirus 2 (SARS-CoV-2), ZIKV, and DENV, vimentin filaments reorganize into cage-like structures to facilitate viral replication by providing a supportive scaffold for viral replication factory (Lei et al., 2013; Cortese et al., 2020; Zhang et al., 2022). While the formation of the vimentin cage is well-documented and its importance in viral lifecycle has been established, strategies to specifically target and disrupt this structure as a potent broad-spectrum anti-viral approach remain unexplored.

The RAS-regulated RAF–MEK1/2–ERK1/2 signaling pathway is a critical cascade in mitogen-activated protein kinase (MAPK) pathway that regulates cell cycle, differentiation, and survival (Cargnello and Roux, 2011; Morrison 2012). This pathway is activated by various extracellular stimuli such as growth factors, cytokines, and pathogens (Klug et al., 2011; Kidger et al., 2018; DuShane and Maginnis, 2019). Notably, many viruses activate MEK1/2–ERK1/2 cascade to boost the viral replication cycle (Bonjardim 2017). For instance, adenovirus and yellow fever virus exploit this signaling cascade to enhance viral genome replication, protein synthesis, and pathogenesis (Schumann and Dobbelstein, 2006; Albarnaz et al., 2014). Consequently, targeting the MEK1/2–ERK1/2 pathway has emerged as a promising anti-viral strategy (Ludwig et al., 2023). Several studies have demonstrated that MEK1/2 inhibitors can effectively suppress viral infections, including notable viruses such as SARS-CoV-2, human immunodeficiency virus type 1 (HIV-1), and influenza A virus (Dochi et al., 2018; Schrader et al., 2018; Schreiber et al., 2020). However, the molecular mechanisms underlying the anti-viral effects of MEK1/2 inhibitors remain unclear.

In this study, we revealed the crucial role and the host target of the MEK1/2 signaling pathway in DENV and ZIKV infections. By employing MEK1/2 inhibitors to mediate vimentin dispersal, along with the dispersion of intracellular viruses, we effectively inhibited the infection of DENV and ZIKV. Therefore, MEK1/2 inhibitors may be harnessed for the specific therapeutic intervention of these two viruses.

## Results

### DENV-2 infection activates MEK1/2 signaling and the inhibition of MEK1/2 compromises DENV-2 infection

**Figure 1:**
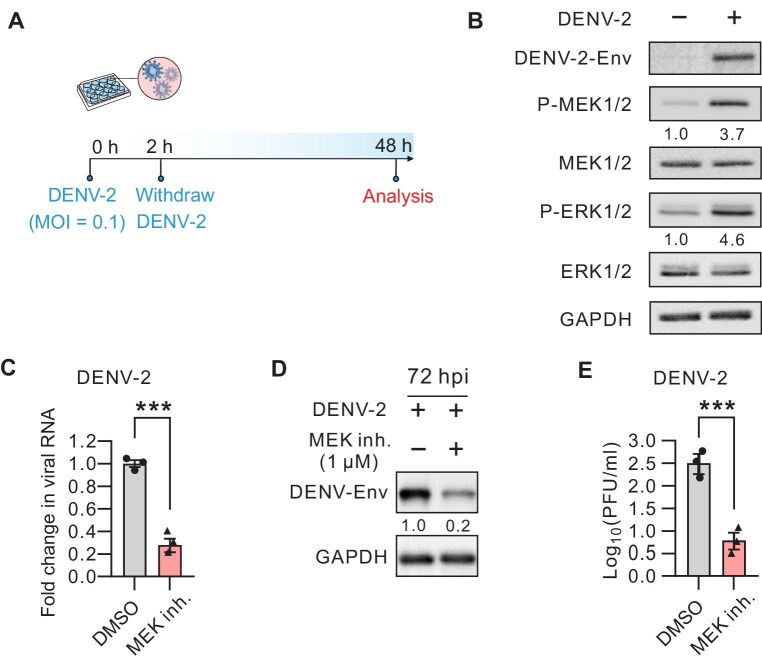
MEK1/2 inhibition suppresses DENV-2 infection. (**A**) Schematic illustration of DENV-2 infection process. (**B**) Western blot analysis of MEK1/2, ERK1/2, and their phosphorylation levels in total cell lysates of U2OS cells at 48 hpi with DENV-2. GAPDH was probed to verify equal sample loading. (**C**) RT-qPCR measurement of intracellular DENV-2 RNA levels relative to GAPDH mRNA in dimethyl sulfoxide (DMSO)- or MEK1/2 inhibitor (Trametinib, 1 µM for 48 h)-treated U2OS cells following 48 h infection with DENV-2 (MOI = 0.1). (**D**) Western blot analysis of DENV-2 envelope (Env) protein levels in the total lysates of DMSO- or MEK1/2 inhibitor (Trametinib, 1 µM for 72 h)-treated U2OS cells following 72 h infection with DENV-2 (MOI = 0.1). GAPDH was probed for equal sample loading. (**E**) The viral yield in the supernatants of DMSO- or MEK1/2 inhibitor (Trametinib, 1 µM for 48 h)-treated U2OS cells following 48 h infection with DENV-2 (MOI = 0.1). Data in **C** and **E** represent the mean ± SEM from three independent experiments. Statistical significance was assessed by unpaired *t*-test (***P < 0.001).

To investigate the host response of DENV infection, we inoculated the highly susceptible human osteosarcoma U2OS cells with one of the variants, DENV-2 (Figure 1A). Western blot analysis indicated that DENV-2 infection significantly enhanced the phosphorylation level of MEK1/2 at 48 h post infection (hpi). Consistent with this observation, phosphorylation of ERK1/2, the downstream effector of MEK1/2, was also markedly elevated in response to DENV-2 infection in U2OS cells (Figure 1B), suggesting that DENV-2 infection triggers the activation of the MEK1/2–ERK1/2 signaling in host cells.

We then hypothesized that MEK1/2 activation is a prerequisite for DENV-2 replication. To evaluate this hypothesis, we exposed DENV-2-infected cells to Trametinib, a specific MEK1/2 inhibitor and a clinical approved drug for curing cancer (Han et al., 2021), immediately after withdrawing the virus and quantified the level of viral replication at 48 hpi. Real-time quantitative polymerase chain reaction (RT-qPCR) analysis revealed a substantial reduction in viral RNA levels in cells treated with Trametinib (Figure 1C). Further assessment of the viral envelope (Env) protein by western blotting and viral titer by plaque assay corroborated the suppressive effects of Trametinib on viral infection (Figure 1D and E). Together, our data demonstrate that DENV-2 infection activates the MEK1/2–ERK1/2 pathway, and pharmacological inhibition of MEK1/2 activity can effectively mitigate DENV-2 infection.

### MEK1/2 inhibitor impedes cytoskeletal vimentin remodeling during DENV-2 infection

**Figure 2:**
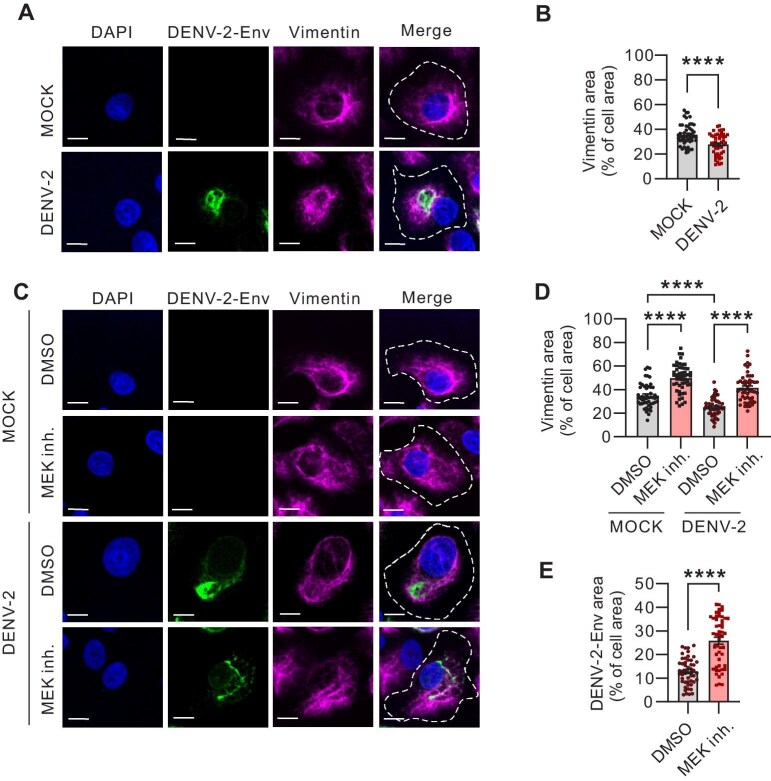
MEK1/2 inhibitor disperses both vimentin network and DENV-2 Env protein. (**A** and **B**) Immunofluorescence images of vimentin network (magenta) and DENV-2 Env protein (green) in U2OS cells at 48 hpi with DENV-2 (MOI = 0.1). White lines represent the cellular outline. Scale bar, 10 µm. The relative vimentin area vs. cell area was quantified. *n* = 100 cells. (**C**–**E**) Immunofluorescence images of the vimentin network (magenta) and DENV-2 Env protein (green) DMSO- or MEK1/2 inhibitor (Trametinib, 1 µM for 48 h)-treated U2OS cells following 48 h of mock DENV-2 infection. White lines represent the cellular outline. Scale bar, 10 µm. The relative vimentin area vs. cell area (**D**) and the relative DENV-2 Env area vs. cell area (**E**) were quantified. *n* = 100 cells. Data in **B, D**, and **E** represent the mean ± SEM from three independent experiments. Statistical significance was assessed using unpaired *t*-test for **B** and **E** or two-way ANOVA test for **D** (****P < 0.0001).

Our previous studies demonstrated that Trametinib, as a MEK1/2 inhibitor, can induce the spatial expansion of the cellular vimentin network without impacting its transcriptional nor translational regulation (Zhao et al., 2023, 2024). In this study, we infected U2OS cells with DENV-2 and visualized the viral Env protein and the endogenous vimentin using immunofluorescence microscopy. In mock-infected cells, vimentin filaments exhibited perinuclear localization and radiated outward toward the cell periphery, presenting a distinct filamentous structure (Figure 2A). Upon DENV-2 infection, the cellular vimentin distribution was significantly altered, forming compact aggregates in close proximity to the viral Env proteins near the nucleus (Figure 2A and B).

Subsequently, we treated the cells with Trametinib. In both mock-infected and DENV-2-infected cells, Trametinib treatment led to a significant expansion of the endogenous vimentin network. This expansion was quantified by measuring the vimentin area relative to the cell area, as depicted in Figure 2C and D. Interestingly, in DENV-2-infected cells, the Env protein also exhibited a diffused distribution following Trametinib treatment, indicating that the viral replication factory also dispersed (Figure 2C and E). Together, our findings suggest that MEK1/2 inhibition by Trametinib impedes the cytoskeletal vimentin remodeling that typically occurs during DENV-2 infection.

### MEK1/2 inhibitor suppresses DENV-2 infection by mediating cytoskeletal vimentin remodeling

**Figure 3:**
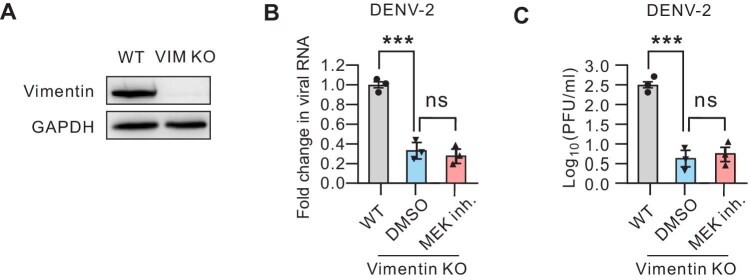
MEK1/2 inhibitor suppresses DENV-2 infection by regulating vimentin in host cells. (**A**) Western blot analysis of endogenous vimentin levels in total cell lysates of wild-type (WT) and vimentin KO U2OS cells, respectively. GAPDH was probed to verify equal sample loading. (**B** and **C**) WT and vimentin KO U2OS cells were infected by DENV-2 (MOI = 0.1) for 48 h and treated with DMSO or MEK1/2 inhibitor (Trametinib, 1 µM for 48 h). (**B**) RT-qPCR was performed to determine the DENV-2 RNA levels normalized to GAPDH mRNA. (**C**) Plaque assay was performed to determine the virus yield in the supernatants. Data in **B** and **C** represent the mean ± SEM from three independent experiments. Statistical significance was assessed by two-way ANOVA test (***P < 0.001; ns, not significant).

Having established that MEK1/2 inhibition can alter the cellular distribution of the vimentin network in DENV-2-infected U2OS cells, we next aimed to determine whether the anti-viral effects of MEK1/2 inhibition are mediated through vimentin. To this end, we generated vimentin knockout (KO) U2OS cells (Figure 3A) and infected these vimentin-deficient cells with DENV-2. RT-qPCR analysis of viral genome replication revealed a significant decrease in viral RNA copies in vimentin KO cells at 48 hpi (Figure 3B). Furthermore, the virus titer assay indicated that the vimentin-depleted cells released ∼80% fewer viral particles at 48 hpi (Figure 3C). Notably, treatment with MEK1/2 inhibitor did not exhibit additional inhibitory effects on DENV-2 replication in the vimentin-deficient cells (Figure 3B and C). Collectively, these findings suggest that MEK1/2 inhibitor-mediated suppression of DENV-2 infection is dependent on the vimentin network.

### MEK1/2 inhibitor suppresses ZIKA infection by mediating cytoskeletal vimentin remodeling

**Figure 4:**
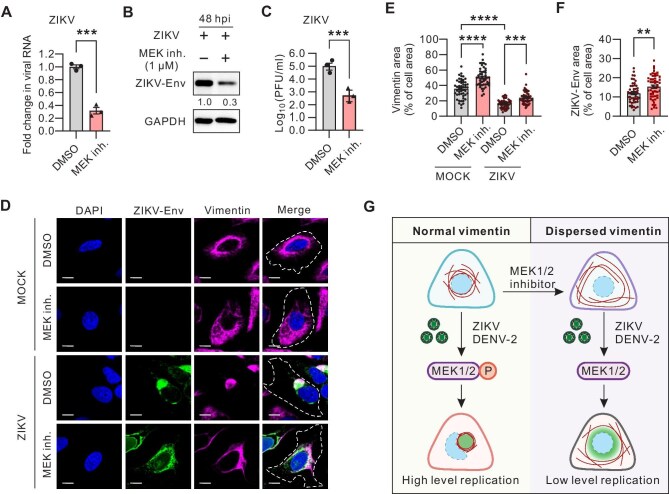
MEK1/2 inhibitor disrupts ZIKV infection by dispersing vimentin network. (**A**) RT-qPCR measurement of intracellular ZIKV RNA levels relative to GAPDH mRNA in U2OS cells infected by ZIKV (MOI = 0.1) for 48 h. (**B**) Western blot analysis of ZIKV Env protein levels in the total lysates of DMSO- or MEK1/2 inhibitor (Trametinib, 1 µM for 48 h)-treated U2OS cells following 48 h infection with ZIKV (MOI = 0.1). GAPDH was probed for equal sample loading. (**C**) The viral yield in the supernatants of DMSO- or MEK1/2 inhibitor (Trametinib, 1 µM for 48 h)-treated U2OS cells following 48 h infection with ZIKV (MOI = 0.1). (**D**–**F**) Immunofluorescence images of the vimentin network (magenta) and ZIKV-Env (green) in DMSO- or MEK1/2 inhibitor (Trametinib, 1 µM for 48 h)-treated U2OS cells following 48 h of mock or ZIKV infection. White lines represent the cellular outline. Scale bar, 10 µm. The relative vimentin area vs. cell area (**E**) and the relative ZIKV Env area vs. cell area (**F**) were quantified. *n* = 100 cells. (**G**) Schematic diagram showing that MEK1/2 inhibitor can suppress the infection of DENV-2 and ZIKV by dispersing vimentin. Data in **A, C, E**, and **F** represent the mean ± SEM from three independent experiments. Statistical significance was assessed using unpaired *t*-test for **E** or two-way ANOVA test for **A, C**, and **F** (**P < 0.01; ***P < 0.001; ****P < 0.0001).

Previous research highlighted the supportive role of the vimentin network in ZIKV infection (Zhang et al., 2022). To explore the potential regulatory effects of MEK1/2 inhibitor on ZIKV infection, we treated ZIKV-infected cells with Trametinib. RT-qPCR analysis revealed a significant reduction in viral RNA levels in ZIKV-infected cells treated with Trametinib (Figure 4A). Additionally, western blot analysis of the viral Env protein and plaque assay for virus titer further confirmed the inhibitory impact of Trametinib on ZIKV infection (Figure 4B and C). Similar to those observed with DENV-2, ZIKV infection also induced a reorganization of the vimentin network and ZIKV Env protein was significantly expanded following Trametinib treatment (Figure 4D–F).

## Discussion

In this study, we observed an increase in MEK1/2 phosphorylation and a reorganization of the vimentin network in cells following DENV infection. The critical role of MEK1/2 activity and vimentin network remodeling in facilitating DENV infection was confirmed through the use of MEK1/2 inhibitors and the vimentin-deficient cells. A comparable interaction mechanism was identified during ZIKV infection, suggesting a broader role for MEK1/2 in virus–host interactions and expanding our understanding of vimentin’s regulation mechanism in cell biology (Figure 4G).

Previous studies reported the significant anti-bacterial activity of MEK1/2 inhibitors in both *in vitro* and *in vivo* settings (Zhao et al., 2023). Our findings corroborate the supportive role of MEK1/2 in the infection processes of both DENV and ZIKV, thereby extending the potential antiviral applications of MEK1/2 inhibitors.

Studies have demonstrated that the MEK1/2–ERK1/2 signaling pathway is activated during infections caused by various viruses, including SARS-CoV-2 (Suzuki et al., 2021), Ebola virus (Strong et al., 2008), Epstein–Barr virus (Liu and Cohen, 2015), adenovirus (Natarajan et al., 2003), and HIV (Schrager et al., 2002). Additionally, these viruses induce the aggregation of the cytoskeletal intermediate filament protein vimentin during infection (Staufenbiel et al., 1986; Liebowitz et al., 1987; Bhattacharyya and Hope, 2011; Fernández-Ortega et al., 2016; Cortese et al., 2020). Based on these observations, we speculate that MEK1/2 inhibitors may suppress viral infection by promoting the dispersion of the vimentin network and disrupting cytoskeletal structures critical to the viral life cycle. The antiviral effects of MEK1/2 inhibitors have been partially validated at the *in vitro* cellular level. Future research will focus on confirming the antiviral efficacy of MEK1/2 inhibitors *in vivo* and evaluating their potential as therapeutic agents for infectious diseases. This study provides a theoretical foundation for considering MEK1/2 inhibitors as candidates for antiviral therapies.

## Methods

### Cell lines and viruses

Human osteosarcoma (U2OS) cells and African green monkey kidney epithelial (Vero) cells were cultured in Dulbecco’s Modified Eagle Medium (DMEM) (#1055-57-1; Biological Industries) supplemented with 10% fetal bovine serum (FBS) (#10270-106; Gibco) and 1% penicillin and streptomycin at 37°C with 5% CO_2_. C6/36 cells were cultured in Minimum Essential Medium (#11095080; Gibco) supplemented with 10% FBS and 2% non-essential amino acids (#N1250-100; Solarbio) at 28°C with 5% CO_2_. ZIKV strain SZ01 (GenBank: KU866423.2) and DENV-2 strain 16881 (GenBank: U87411) were used in this study. ZIKV was amplified in C6/36 cells at a multiplicity of infection (MOI) of 0.1. DENV-2 was amplified in Vero cells at 37°C with 5% CO_2_. Virus-containing supernatants were harvested on Day 4 post-infection and stored at −80°C.

### Virus infection

U2OS cells and Vero cells were seeded into 12-well plates at a density of 2 × 10^5^ cells/well. The amount of virus to be added was calculated based on the viral titer of DENV or ZIKV and the total number of cells. The virus was diluted in DMEM without FBS to infect the cells for 2 h at 37°C. After 2 h, the medium was replaced with fresh DMEM containing 2% FBS. Subsequent experiments were conducted following a 48-h or 72-h infection period.

### Chemical treatment

For non-infected cells, cells were cultured in plates overnight, rinsed with phosphate-buffered saline (PBS), and the medium was replaced with drug-containing medium. For infected cells, medium was replaced with drug-containing medium after withdrawing the virus. Trametinib (#S2673; Selleck; 1 µM) was used.

### Immunofluorescence microscopy

Cells were seeded in a 24-well plate at a density of 4 × 10^4^ cells per well on coverslips (#631-0150; VWR). The cells were washed with PBS, fixed with 4% paraformaldehyde (PFA) for 15 min at room temperature, washed again with PBS, and permeabilized with 0.1% Triton X-100 in PBS for 5 min. The cells were then blocked with 5% bovine serum albumin (#A23088; ABCONE) in PBS for 30 min, and the following primary antibodies were used: vimentin rabbit monoclonal D21H3 antibody (#5741; Cell Signaling Technology; dilution 1:100); vimentin mouse monoclonal V9 antibody (#ab24525; Abcam; dilution 1:100); ZIKV envelop mouse monoclonal antibody (#1176-46; BioFront; dilution 1:1000); ZIKV NS4B rabbit polyclonal antibody (#GTX133311; Genetex; dilution 1:1000); DENV-2 envelop rabbit polyclonal antibody (#GTX127277; Genetex; dilution 1:2000). The following secondary antibodies were used: Alexa Fluor 488 goat anti-rabbit IgG (H+L) (#A11008; Invitrogen; dilution 1:1000); Alexa Fluor 568 goat anti-rabbit IgG (H+L) (#A11011; Invitrogen; dilution 1:1000); Alexa Fluor 488 goat anti-mouse IgG (H+L) (#A11001; Invitrogen, dilution 1:1000); and Alexa Fluor 555 goat anti-mouse IgG (H+L) (#A21422; Invitrogen; dilution 1:1000). Both primary and secondary antibodies were applied to the cells and incubated at room temperature for 1 h sequentially. F-actin was stained by Alexa Fluor 647 phalloidin (#A22287; Invitrogen; dilution 1:500) to determine the cell outlines. After washed three times with PBS, slices were mounted in DAPI fluoromount-G reagent (#0100-20; SountherBiotech). Imaging data were obtained using Olympus SpinSR10 Ixplore spinning disk confocal microscope with 60× UplanApoOil objective (NA = 1.2, Olympus Corporation).

### Western blotting

Cells were washed with cold PBS and lysed in RIPA lysis buffer (#P0013B; Beyotime) supplied with protease and phosphatase inhibitors (#P1045; Beyotime) on ice for 10 min. Cell debris was removed by centrifugation at 12000× *g* for 10 min at 4°C. Protein concentrations were measured using the BCA kit (#P0012; Beyotime), adjusted with PBS and 6× sodium dodecyl sulfate (SDS)-sample buffer (#P0015F; Beyotime), and subjected to SDS–polyacrylamide gel electrophoresis. Proteins were transferred to polyvinylidene fluoride membranes (#IPVH00010; Millipore) and blocked with protein-free rapid blocking buffer (#PS108P; Epizyme Biotech) at room temperature for 15 min. Membranes were incubated with primary antibodies overnight at 4°C. The following primary antibodies were used: vimentin rabbit monoclonal D21H3 antibody (#5741; Cell Signaling Technology; dilution 1:1000); p44/42 MAPK (ERK1/2) (137F5) rabbit monoclonal antibody (#4695; Cell Signaling Technology; dilution 1:1000); phospho-p44/42 MAPK (ERK1/2) (Thr202/Tyr204) (D13.14.4E) rabbit monoclonal antibody (#4370; Cell Signaling Technology; dilution 1:1000); MEK1/2 (L38C12) mouse monoclonal antibody (#4694; Cell Signaling Technology; dilution 1:1000); phospho-MEK1/2 (Ser217/221) (41G9) rabbit monoclonal antibody (#9154; Cell Signaling Technology; dilution 1:1000); ZIKA envelop mouse monoclonal antibody (#1176-46; BioFront; dilution 1:5000); DENV envelop rabbit polyclonal antibody (#GTX127277; Genetex; dilution 1:2000); and GAPDH rabbit monoclonal antibody (#G8795; Sigma–Aldrich; dilution 1:5000). Membranes were washed with Tris-buffered saline containing 0.1% Tween20 four times for 10 min each and incubated with secondary antibodies for 1 h at room temperature. Horseradish peroxidase (HRP)-linked anti-mouse IgG antibody (#7076 V; Cell Signaling Technology; dilution 1:5000) and HRP-linked anti-rabbit IgG antibody (#7074 V; Cell Signaling Technology; dilution 1:5000) were used. Chemiluminescence was detected using Chemistar High-sig ECL Western Blotting Substrate (#180-501; Tanon). Band intensities of blots were measured with ImageJ software, and protein levels were normalized to GAPDH.

### RT-qPCR

Total cellular RNA was extracted using EZ-press RNA Purification Kit (#B0004DP; EZBioscience) according to the manufacturer’s instructions. Reverse transcription of total RNA was performed using Color Reverse Transcription Kit (#A0010CGQ; EZBioscience). RT-qPCR was conducted with 2× Color SYBR Green qPCR Master Mix (ROX2 plus) (#A0012-R2; EZBioscience) in QuantStudio 6 system (Thermo). All readings were normalized to the level of GAPDH. The following primers were used: ZIKV-forward, 5′-CAACCACAGCAAGCGGAAG-3′; ZIKV-reverse, 5′-AAGTGATCCATGTGATCAGTTGATCC-3′; DENV-2-forward, 5′-GGTTAGAGGAGACCCCTCCC-3′; DENV-2-reverse, 5′-GAGACAGCAGGATCTCTGGTCT-3′; GAPDH-forward, 5′-GCATCCTGCACCACCAACTG-3′; and GAPDH-reverse, 5′-GCCTGCTTCACCACCTTCTT-3′. Results were expressed as the fold differences, with vehicle-treated cells used as the calibrator value.

### Virus plaque assay

DENV-2 titers were determined by focus-forming assay. In brief, 1 × 10^4^ Vero cells were seeded in a 96-well plate and incubated overnight at 37°C. Cells were then infected with serially diluted DENV-2 supernatants for 2 days. The cells were fixed with 4% PFA and incubated with antibody against DENV envelop protein (#GTX127277; Genetex; dilution 1:2000), followed by Alexa Fluor 488-conjugated secondary antibody and Hoechst 33258. The stained cells were analyzed using fluorescence microscopy.

ZIKV titers were determined by plaque assay on Vero cells as previously described (Zhang 2022 PNAS). Briefly, 1 × 10^4^ Vero cells were seeded in 24-well plates and incubated for 20 h. The cells were washed twice with PBS and infected with serially diluted ZIKV for 2 h. The inoculum was then replaced with 1 ml DMEM containing 1.5% FBS and 1% carboxymethylcellulose (#C5678; Sigma). On Day 4 post-infection, the cells were fixed with 4% PFA and stained with crystal violet (#C0121; Beyotime) for 10 min to visualize plagues. Viral titers were expressed as plaque-forming units (PFU) per milliliter.

### Image analysis

Fluorescence images were processed and analyzed using Fiji software (http://fiji.sc). Cell areas were quantified by defining the cell edge through the outer face of peripheral actin staining. Vimentin filaments were mapped using the Ridge Detection plugin.

### Statistical analysis

Statistical analysis was performed by unpaired *t*-test, one-way analysis of variance (ANOVA) test, or two-way ANOVA test using the GraphPad Prism v9 software, and *P*-values were indicated by ns (*P* > 0.05), *P < 0.05, **P < 0.01, ***P < 0.001, and ****P < 0.0001. The histogram data were presented as mean ± SEM.
